# The Theoretical and Methodological Opportunities Afforded by Guided Play With Young Children

**DOI:** 10.3389/fpsyg.2018.01152

**Published:** 2018-07-17

**Authors:** Yue Yu, Patrick Shafto, Elizabeth Bonawitz, Scott C.-H. Yang, Roberta M. Golinkoff, Kathleen H. Corriveau, Kathy Hirsh-Pasek, Fei Xu

**Affiliations:** ^1^Department of Psychology, Rutgers University-Newark, Newark, NJ, United States; ^2^Department of Mathematics and Computer Science, Rutgers University-Newark, Newark, NJ, United States; ^3^School of Education, University of Delaware, Newark, DE, United States; ^4^School of Education, Boston University, Boston, MA, United States; ^5^Department of Psychology, Temple University, Philadelphia, PA, United States; ^6^Department of Psychology, University of California, Berkeley, Berkeley, CA, United States

**Keywords:** guided play, computational modeling, data science, direct instruction, free play

## Abstract

For infants and young children, learning takes place all the time and everywhere. How children learn best both in and out of school has been a long-standing topic of debate in education, cognitive development, and cognitive science. Recently, *guided play* has been proposed as an integrative approach for thinking about learning as a child-led, adult-assisted playful activity. The interactive and dynamic nature of guided play presents theoretical and methodological challenges and opportunities. Drawing upon research from multiple disciplines, we discuss the integration of cutting-edge computational modeling and data science tools to address some of these challenges, and highlight avenues toward an empirically grounded, computationally precise and ecologically valid framework of guided play in early education.

## Introduction

Learning in school is often characterized by structured courses and tasks with discrete and explicit objectives. Yet, learning is a continuous process that also takes place outside the classroom where explicit objectives are not always evident. This is especially true in early childhood interactions at home, where children often learn from everyday interactions with both the physical environment and with social partners (Bruner, 1961; Csibra and Gergely, 2009). How to best navigate between explicit, objective-directed learning and more flexibly driven exploration has been a longstanding topic of debate in education, developmental psychology, and cognitive science (Kirschner et al., 2006; Tobias and Duffy, 2009). This debate surfaces in a number of forms, as direct instruction vs. discovery learning or as work vs. play (Bonawitz et al., 2011; Hirsh-Pasek and Golinkoff, 2011; Clements and Sarama, 2014). Pitting these two interests against each other has neither optimized our understanding of learning, nor produced optimal methods of learning (Wise and O’Neill, 2009). Here, we discuss an integrated approach, *guided play*, that enables us to rethink learning as a child-led, adult-assisted activity (Weisberg et al., 2013, 2014, 2016). Focusing on everyday interactions in early childhood, guided play is operationally defined as learning that is active and engaged, where the child takes initiative in a playful learning environment and the adult supports, rather than directs, the learning experience. Sitting between free play, where children explore by themselves, and direct instruction, where the interaction is led by an adult and children take a passive role, guided play takes advantage of the latest research in the science of learning.

Educational research indicates that student-led discovery learning that is facilitated by teachers outperforms both direct instruction and unassisted discovery (Mayer, 2004; Honomichl and Chen, 2012). In a meta-analysis comparing explicit instruction, unassisted discovery, and assisted discovery (Alfieri et al., 2011), learning outcomes were more favorable for assisted discovery than for other forms of instruction. These results held for learners of different ages and across different learning domains. Similarly, developmental studies have shown an advantage of adult guidance over both direct instruction and free play, even before children start formal schooling (Han et al., 2010; Fisher et al., 2013; Ridge et al., 2015; Haden et al., 2016; Sim and Xu, 2017; Yu et al., 2018). In both bodies of literature, “guidance” has referred to a variety of practices including modeling, questioning, encouragement, and feedback, and thus it is unclear what particular aspects of guidance are associated with learning (Wise and O’Neill, 2009; Honomichl and Chen, 2012).

In *guided play*, learning opportunities may be explicitly structured, but importantly the activity is child-led. Specifically, we define “*guidance*” as adults’ involvement that subtly channels the dyadic interactions to fulfill certain pedagogical objectives, while not interfering too much so that the activities remain child-led. The pedagogical objectives can be multi-level: they can focus on specific content knowledge, but can also focus on the emotional, motivational, and metacognitive aspects of the learning process, such as cultivating children’s love of learning, promoting their engagement, or making them aware of their own learning process (Weisberg et al., 2014). Our concept of guidance is inspired by the Vygotskian concept of scaffolding (Vygotsky, 1934/1987; Wood et al., 1976; Fernández et al., 2001) and Barbara Rogoff’s theory of guided participation (Rogoff et al., 1993). In addition to guidance being tailored to fit individual children’s needs and skill level (which is similar to scaffolding), in guided play we also emphasize that guidance should never shift children away from controlling their own learning process. The pedagogical objectives of guidance are therefore broader—besides helping children to master particular knowledge or skills, guided play also aims to provide children with an opportunity to enjoy, control, and reflect upon their own learning process, which may facilitate independent inquiry and discovery in the future.

Because guided play requires seamless integration between the adult’s objectives to support learning and child-led activity that can be highly fluid, characterizing appropriate guidance requires an understanding of the dynamic nature of an adult–child interaction in context. First, guided play is *interactive.* How well-children can learn from a playful interaction depends on their mental state at the moment—including their level of knowledge, goal, attention, emotion, trust toward the play partner, etc. Therefore, effective guidance should take into account and be contingent upon the mental state of the child. This requires theories to consider the dyad as a system moving toward a joint objective (Fogel and Garvey, 2007; Lavelli et al., 2015; Heller and Rohlfing, 2017), and requires experimental designs and analytical tools that go beyond between-group comparisons to focus on individual dyads. Second, guided play is *dynamic*. Timing is critical for the guidance to be effective. Providing a label, for example, can be educational at a moment when a child is focusing on the target object, but can be confusing when the child is focusing on multiple objects (Pereira et al., 2014). Similarly, demonstrating object functions when an infant is pointing to the object also supports learning (Begus et al., 2014). For preschoolers, revealing causal features of objects right before, but not after, a demonstration of categorization facilitates children’s category learning (Yu and Kushnir, 2016). Existing theories, such as direct instruction and free play, and methodological tools, such as standard statistical tests, are optimized for discrete interventions and are usually applied uniformly across groups of individuals. Characterizing the dynamic nature of guided play will require development of new theories and tools to capture interventions along a continuous timeline. In what follows, we detail these theoretical and methodological matters, the tools that may be used to address them, and the prospects for a theory of guided play.

## Theoretical Challenges and Opportunities for Guided Play

Free play and direct instruction have long been contrasted in education and cognitive development (Dewey, 1933; Mayer, 2004; Kirschner et al., 2006; Hirsh-Pasek et al., 2008), and existing mathematical and computational models for the two scenarios have likewise been developed separately because they typically focus on different aspects of learning (Nelson, 2005; Shafto et al., 2014). Free play is based on the constructivist views of learning, which portrays learning as an active process during which the learner repeatedly intervenes on their environment, and updates their beliefs based on information gathered from these experiences (Piaget, 1952). Correspondingly, computational models of free play have largely focused on how to sequentially choose evidence during learning (Nelson, 2005; Settles, 2010; Markant and Gureckis, 2014; McCormack et al., 2016). These models generate predictions about how the optimal next step will depend on the current state and are therefore *dynamic*. However, such models are inadequate to capture the *interactive* aspect of guided play because they do not usually simulate a social partner whose behavior is contingent on the learner.

In contrast, direct instruction emphasizes the necessity of outside instructions for learners to successfully navigate a learning task (Kirschner et al., 2006), and focuses on what content should be delivered by instruction (Mayer, 2004). Correspondingly, computational models of direct instruction have focused on the evidence teachers should select to lead learners to the correct answer, given the learner’s current beliefs (Shafto and Goodman, 2008; Shafto et al., 2012b, 2014; Frank, 2014; Zhu, 2015; Rafferty et al., 2016). Some of these models simulate the *interactive* nature of teaching and learning through modeling the teacher and the learner’s reasoning about the other’s knowledge levels and objectives (Shafto and Goodman, 2008; Shafto et al., 2012b, 2014). However, these models are not *dynamic*; they select evidence with the immediate goal of the learner arriving at the correct inference. When dynamic extensions have been proposed, they encounter significant computational challenges that render the models of limited use for modeling real-life scenarios (Rafferty et al., 2016; Yang and Shafto, 2017).

Theories and models of epistemic trust may inform modeling of dynamic interactions between a teacher and a learner. The literature on epistemic trust has investigated the dynamics of reasoning, focusing on a learner’s sensitivity to both a teacher’s prior knowledge in a given domain (Pasquini et al., 2007; Sobel and Corriveau, 2010) as well as her social group membership when making decisions about whom to trust (Kinzler et al., 2011; Chen et al., 2013). Models of epistemic trust (Eaves and Shafto, 2012, 2017; Shafto et al., 2012a) tend to build upon aforementioned models of direct instruction. Although both of these bodies of work make the prediction that children’s epistemic and social evaluation of a teacher should influence their trust in her (and therefore, their sensitivity to her guidance), to date, both the experimental and computational work has focused on the dynamics of trust, but not learning.

Finally, ecological psychology and dynamic systems approaches have been applied to analyze dynamic interactions between adults and children (Bronfenbrenner, 1986; Thelen and Smith, 1996; Fogel and Garvey, 2007). These approaches were foundational in emphasizing the need to view adult–child interactions as a system that evolves through time, as well as the need to situate these interactions in the immediate environment. They also provided invaluable computational tools to analyze patterns of co-activities that emerges along time. Because formal dynamic systems models often focus on overt behavior, applying these models to guided play may require an extension which takes into account the mental state and inferential capacities of both learners and guiding adults.

A unified theory of guided play must combine strengths from previous research to capture the interactive and dynamic nature of learning. A key challenge for proposing such a theory is the development of theoretical frameworks that avoid simulating every possible mental state of the teacher and the learner, which would create intractable computational problems. Even the simplest learning situations involve many potential choices by both learners and guiding adults. For example, when an adult guides a child to learn the name of an object, the adult could choose from a variety of actions (e.g., pointing to the object, holding it, looking at the child, or looking at the object) as well as utterances (e.g., naming the object, or asking a question), and the child could also respond in a variety of ways (e.g., reaching for the object, repeating the word, or displaying a puzzled face). Adults and children nevertheless navigate such situations, making choices while balancing short- and long-term objectives. To simulate these capacities, one approach is to adopt simplified computational models similar to those employed in the educational technology literature. One example is Bayesian knowledge tracing, which instead of modeling the learner’s full belief state, focuses on whether the learner has the correct concepts (Corbett and Anderson, 1995; Yudelson et al., 2013). A second approach is to use task-specific information to limit the set of relevant actions. For example, an approach that pairs observation of naturalistic adult–child interaction during a task with an experiment that measures the learning outcome of that task could help to identify the task-relevant subset of information (Yu et al., 2017). Subsequent experimental studies could then test predictions of the model on this reduced set of relevant information rather than the whole set of logical possibilities.

## Methodological Challenges and Opportunities for Guided Play

The interactive and dynamic properties of guided play also pose questions for experimental design and analysis that may require modifications of existing tools and the development of new ones. One source of methodological challenges arises from variations in the effectiveness of guidance based on individual characteristics of the child. Guidance *content* that is effective for one child may not be effective for a different child. For example, two children may have different misconceptions about what constitutes a triangle (Fisher et al., 2013). One may think a triangle needs to have the point at the top, whereas the other may think a triangle needs to have all acute angles. In this case, different examples should be presented to guide these two children away from their respective misconceptions: it would be more effective to show the first child a real triangle with point in the bottom, and show the second child an obtuse triangle. This intuition is supported by research: research in category learning has shown that a set of evidence that is effective in facilitating one person’s learning may be less effective when presented to another person (Markant and Gureckis, 2014; Sim et al., 2015). In addition, individual differences in children’s background knowledge, cognitive style, and experiences with different sociocultural practices can all influence the effectiveness of presenting certain content to them (McNamara et al., 1996; Gutiérrez and Rogoff, 2003; Price, 2004). Individual differences remain an important topic for further research.

The *timing* of guidance is also important: well-timed guidance that is contingent upon the child’s prior actions may impact child learning outcomes differently than if the same guidance is not well-timed (Pereira et al., 2014). Such variability in guidance content and timing poses challenges to typical random-assignment controlled experiments, as uniform interventions applied to groups of randomly assigned individuals do not necessarily test the interactive and dynamic predictions of guided play. Yet observational designs are insufficient to tease apart the causal relations between components of guided play and children’s learning outcomes. Therefore, new methods and analytical tools are required to select the content and timing of guidance to maximally inform our understanding of the mechanisms involved in guided play.

Advances in data science and technology may provide tools for addressing some of these challenges by providing an opportunity for real-time analysis and feedback, as well as (semi-)automatic analysis of large amounts of time series data. For example, in word-learning scenarios, children look at the experimenter more when they are uncertain about an object label (Hembacher et al., 2017). Thus, an overt behavior, here eye gaze, reveals important information about the learner’s mental state, and could represent opportunities for guidance. Technological advances in eye-tracking equipment and data sharing mechanisms have allowed for the collection and sharing of large-scale, live-stream video data from naturalistic adult–child interactions (Franchak et al., 2011; Databrary, 2012). However, coding and analysis of children’s looks are usually conducted manually, which restricts the amount of data that can be utilized and precludes real-time feedback during the interaction. Applying tools of automatic decoding of eye movements and looking, such as those used in vision research (Duc et al., 2008; Gottlieb et al., 2013; Borji and Itti, 2014), may allow for the online recognition of the referent associated with the child’s gaze, which, in turn, may help to nominate a range of appropriate guidance “moves” that are contingent upon the child’s attention and mental state. Indeed, research in social robotics has implemented gaze and action detection in robot *learners* to infer human teachers’ pedagogical intent based on their gaze and actions, and to react in a contingent way (e.g., when the teacher showed an object with pedagogical cues, the robot turned head to the same object; then when the teacher looked back at the robot’s eyes and labeled the object, the robot looked at the teacher and smiled). Human teachers were more engaged and more likely to attribute human-like traits to the robot when the robot displayed these contingent reactions (Lohan et al., 2012). Similar algorithms may also support *teachers* who provide guidance contingent on the learner’s behavior.

Similarly, the learner’s affect and engagement play an important role (Greene and Noice, 1988; Rader and Hughes, 2005). In guided play, the joy that accompanies play helps to sustain motivation, interest, and excitement, which should be associated with enhanced learning outcomes (Hirsh-Pasek and Golinkoff, 2003; Weisberg et al., 2016). Unfortunately, given the time-intensive nature of affect coding, the evidence relating affective states to improved learning outcomes is less extensive. Data science tools may be used to automatically identify affect and engagement in real-time video streams for analysis, and to time guidance to foster affect that predict positive short term and long term learning (Littlewort et al., 2006; Yao et al., 2015; Baker et al., 2017). Such analytical tools would allow for direct tests of guided play predictions related to the timing of learning, while employing experimental designs that are similar to those typically used in the developmental and educational literature.

## Coupling Computational Models and Data Science Tools

A more ambitious possibility is to couple models and data science tools to create experiments highlighting times when interventions may yield the strongest test of the theory. Attempts at interactive, dynamic approaches to teaching can be found in the literature of social robotics and intelligent tutoring systems (Anderson et al., 1985; Breazeal, 2002; Thomaz and Breazeal, 2008; Lohan et al., 2012; Nguyen and Oudeyer, 2014; Vollmer et al., 2014; Clement et al., 2015), in which data from expert teachers have been used to train algorithms to learn the contingencies between learner’s behavior and teachers’ appropriate response (Ruvolo et al., 2008). Such data-driven approaches can serve as a first step for identifying patterns in guided-play interactions. However, to understand characteristics of effective guidance, we also need theory-driven computational models that can represent children’s mental states based on their behavior. Such models differ from existing intelligent tutoring systems in that instead of teaching knowledge in specific domains, they are designed to understand the general principles of effective guidance in a wide range of child-led activities that may or may not have an explicit learning goal. Coupling such models with empirical data could inform an algorithm that predicts appropriate guidance based on children’s behavior, which could in turn be used in experiments to verify the effect of guidance on children’s learning. These experiments would have significant advantages relative to classic training studies, as the intervention is based on an online algorithm which would adapt based on children’s moment-by-moment behavior.

Consider how such computational models could be applied to a recent study of guided play (Fisher et al., 2013). This study examined different pedagogical methods on preschoolers’ learning of geometric shapes, with increased learning in guided play as compared to didactic instruction and free play. In the guided play condition, the experimenter presented two typical examples (e.g., upright triangles) and two atypical examples (e.g., inverted triangles) in a playful manner, and asked children to determine what makes them the same shape. During children’s active exploration the experimenter used questions, encouragement, and feedback to guide them toward the correct answer. Yet, because the interaction was dynamic, the manner and timing of adult guidance were not prespecified in the experimental design, which makes it difficult to pinpoint what aspects of guidance resulted in the enhanced learning outcomes.

**FIGURE 1:**
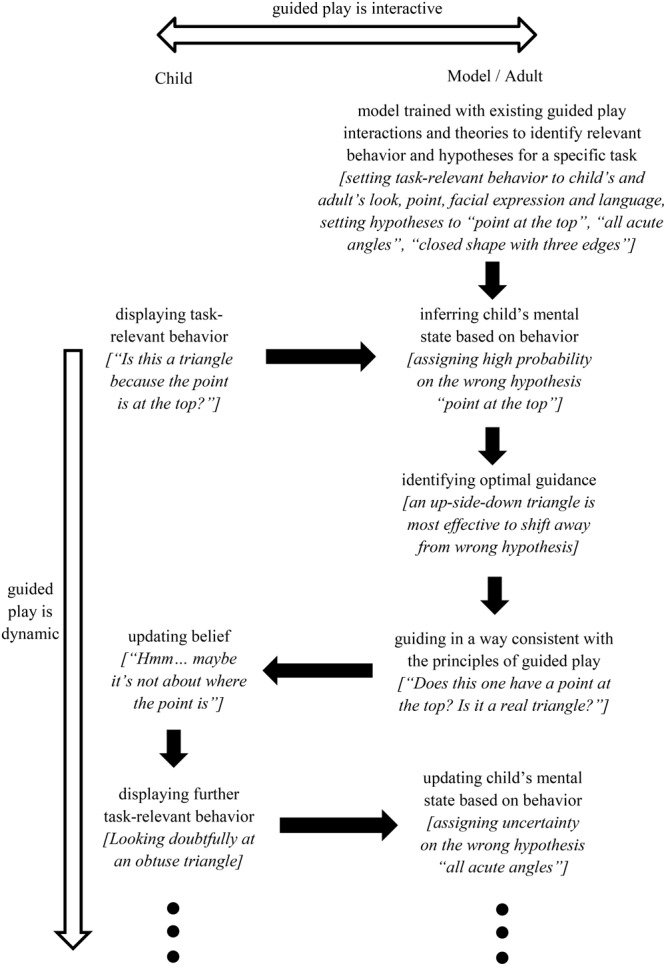
We propose a framework that integrates computational modeling and data science to address challenges brought by the interactive and dynamic nature of guided play. By modeling children’s moment-to-moment mental state from their task-relevant behavior, the proposed framework identifies guidance that are optimized in terms of timing and form, with the objective of sustaining the children’s interests toward the learning goal. The italic text provides an example of learning geometric shapes (Fisher et al., 2013) to show how the framework could be implemented to a specific guided play interaction. This framework can facilitate research of guided play by identifying key aspects of guidance within the dynamic and complex interactions children experience in their everyday environment.

Following the aforementioned framework, existing videos of guided play interactions could be used to train a computational model of learning geometric shapes in four steps (**Figure 1**): first, data science tools can identify a set of common task-relevant behavior during children’s active exploration, and cluster behaviors into categories (e.g., children’s looking and pointing may be categorized as seeking guidance from the experimenter; their emotion as confident vs. doubtful; their language as statements or questions). Tools of this stage could build upon advances in (semi-)automatic recognition of eye gaze (e.g., Lohan et al., 2012; Smith et al., 2015), emotion (e.g., Baker et al., 2017), natural language including information-seeking questions (e.g., Rothe et al., 2016), among others.

Second, a computational model can be used to simulate children’s moment-to-moment beliefs about geometric shapes based on these behavioral patterns. For example, if children point to an upright triangle, look doubtfully at the experimenter, and ask “Is this a triangle because the point is at the top?,” their presumed belief about triangles would shift toward the wrong hypothesis of “point at the top,” with a flat distribution indicating uncertainty. The model at this stage could be built upon existing work that links behavior with mental states on a microgenetic scale, including those that model shifting hypotheses (e.g., Bonawitz et al., 2014), epistemic trust (e.g., Eaves and Shafto, 2017), and automatic goal inference (inverse reinforcement learning; e.g., Baker et al., 2009).

Third, a model of guidance can identify the most effective intervention given children’s current belief. For example, in the aforementioned scenario, to shift children’s belief away from the wrong hypothesis and toward the correct hypothesis, the best example to show may be a real triangle with the point at the bottom. Existing models of teaching, such as the model presented in Rafferty et al. (2016), has used partially observable Markov decision process to optimize teaching actions given the learner’s observed behaviors as well as previous teaching actions. Similar approaches could be used to build models that optimize guidance based on children’s current belief. Importantly, the model is not intended to immediately lead the child to the correct hypothesis as in direct instruction (e.g., “Triangles are shapes bounded by three edges and three vertices”), rather it optimizes the child’s interest to guide them toward the correct hypothesis. In this way, guided play remains child-led.

Finally, the recommended intervention can be carried out by the experimenter in a way that is consistent with the principles of guided play (e.g., through questions like “What about this one [pointing to the inverted triangle]? Does it have point at the top? Is it a real triangle?”).

Once trained, this model will significantly advance our understanding of (1) how individual children grasp concepts of geometric shapes; (2) common misconceptions along the way; and (3) optimal interventions. The resulting model-based interventions allow for guidance tailored to the learner’s moment-by-moment belief states.

## Prospects and Directions for a Theory of Guided Play

For children, learning takes place everywhere, all the time, and often involves interactions by the learner with more knowledgeable individuals. This ubiquity of learning opportunities can be exploited by providing subtle guidance that is contingent on the environment and children’s current mental state (Ridge et al., 2015). Although research has highlighted the advantage of guided play, as compared to direct instruction or free play for facilitating learning (Alfieri et al., 2011; Fisher et al., 2013; Haden et al., 2016; Sim and Xu, 2017; Yu et al., 2018), pinpointing the optimum content and timing of guidance requires an understanding of the interactive and dynamic nature of an adult–child interaction.

We suggest that integrating computational models and data science tools may help lay out an avenue toward an empirically grounded and computationally precise framework of guided play. *By modeling children’s moment-to-moment mental state and the responsive behavior from adults, the proposed model has the potential to identify different components of guided play from dynamic and individualized interactions, and recommend model-based interventions that are optimized in terms of timing and form, with the objective of sustaining the child’s interests toward the learning goal.* The resulting theory of guided play could identify key aspects of guidance that makes guided play effective in a particular context, while maintaining the complexity and ecological validity that comes with the interactive and dynamic nature of the theory. The goal is to use this framework to understand how learning proceeds and when it succeeds, which will also depend on the cultural context and individual learner. Future work could further extend the framework from one-on-one interactions in early childhood to more complex learning scenarios and topics, such as those in a classroom setting. We hope such a framework will shed light on principles of optimal environments and practices to facilitate children’s learning, and present an example of using new approaches to studying cognitive development.

## Author Contributions

YY, PS, and EB drafted the manuscript. All authors were involved in editing the manuscript.

## Conflict of Interest Statement

The authors declare that the research was conducted in the absence of any commercial or financial relationships that could be construed as a potential conflict of interest. The handling Editor is currently co-organizing a Research Topic with one of the authors, KH-P, and confirms the absence of any other collaboration.
